# Analysis of Opinion Evolution Based on Hegselmann–Krause Model with Historical Opinion

**DOI:** 10.3390/e28050541

**Published:** 2026-05-10

**Authors:** Yuqi Zhou, Junyao Sun

**Affiliations:** School of Computer and Artificial Intelligence, Beijing Technology and Business University, Beijing 100048, China

**Keywords:** opinion dynamics, historical opinion, social networks, bounded confidence model, Hegselmann–Krause model

## Abstract

In realistic social networks, individuals are influenced not only by current interactions, but also by recent historical opinions, prior experience, and external guidance. However, historical dependence and its decaying effect remain insufficiently studied in bounded-confidence opinion dynamics. To address this issue, this paper proposes an extended Hegselmann–Krause (HK) model in which each individual updates its opinion according to four information sources: the current opinion, historical opinions, neighbors’ opinions, and a target opinion. The historical-opinion term is modeled as a weighted average of recent historical opinions, and its influence is regulated by an attenuation rate to capture memory decay over time. Simulation experiments are conducted to examine the effects of confidence thresholds, attenuation rates, weighting coefficients, and network topology on opinion evolution. The results show that low confidence thresholds tend to generate fragmented clusters, moderate thresholds facilitate opinion integration, and excessively high thresholds may lead to rapid homogenization. The attenuation rate regulates the balance between historical dependence and adaptability to new information, while different weighting configurations produce distinct evolution patterns. In addition, comparisons across ER random, WS small-world, and BA scale-free networks show that network topology significantly affects convergence speed and final opinion distributions. Finally, simulations on a real-world review-network topology derived from the Epinions dataset illustrate the applicability of the proposed model in an e-commerce-related setting. These findings extend the HK framework from a memory-aware perspective.

## 1. Introduction

Opinion dynamics is an important topic for understanding how opinions evolve in social networks and has attracted increasing attention in recent years [1]. With the rapid development of information technology, individuals interact through increasingly heterogeneous social networks, and collective patterns such as consensus, polarization, and fragmentation may gradually emerge. To study such processes, several classical opinion dynamics models have been proposed, including the DeGroot model [2], the Deffuant–Weisbuch (DW) model [3], the Hegselmann–Krause (HK) model [4], and the continuous opinion and discrete action (CODA) model [5].

Among them, the HK model has been widely studied because it captures an important feature of real opinion interaction, namely that individuals are more likely to be influenced by others whose opinions are sufficiently close to their own. This bounded-confidence line of research has been systematically reviewed in recent studies [6]. Many extensions of HK-type models have been proposed by considering group pressure [7], social conformity [8], heterogeneous confidence thresholds [9], extremist opinions [10], and other bounded-confidence mechanisms [11]. Related studies have also discussed social influence from a broader theoretical perspective [12,13], while heterogeneous bounded-confidence interaction beyond the HK framework has also been examined [14]. In addition, some studies introduce opinion leaders or influential agents into bounded-confidence settings and examine their effects on group polarization [15], consumer preferences in e-commerce networks [16], and information diffusion in competing advertising scenarios [17]. Other studies focus on the discrepancy between expressed opinions and private beliefs, including the basic expressed-private framework [18], its extension with heterogeneous stubbornness [19], and further developments incorporating behavioral inference and conformity constraints [20]. Increasing attention has also been paid to psychological and cognitive mechanisms, such as cognitive dissonance [21], backfire effects and biased assimilation [22], self-persuasion [23], attitude hiding [24], and the combined effects of cognitive dissonance and introversion on echo chamber formation [25].

More broadly, the literature on opinion dynamics has also examined several related directions beyond the standard bounded-confidence setting. The integration of multiple sources of influence has been recognized as an important issue in opinion dynamics [26]. Some recent studies have further considered higher-order bounded-confidence interaction [27] and collective information acquisition [28]. Memory-related and path-dependent opinion evolution has also been explicitly investigated in recent work [29,30]. In addition, recent studies have explored the co-evolution of opinions and interpersonal ties, indicating that polarization or related collective patterns may emerge together with changes in network relations and structural balance [11]. Taken together, these studies suggest that temporal dependence, enriched updating mechanisms, and evolving interaction structures have attracted increasing attention in the literature.

Despite these advances, there is still room for further study on one important aspect of realistic opinion formation: individuals are often influenced not only by current interactions, but also by their own recent historical opinions. In many real-life situations, people do not update their views solely according to the opinions currently observed from neighbors. Instead, judgments are also shaped by prior experiences, previously formed attitudes, and the inertia of earlier cognitive states. Moreover, the influence of such historical opinions is usually not constant. Rather, it tends to decay over time as new information accumulates. Previous HK-type extensions have enriched the modeling of social influence, public–private divergence, psychological mechanisms, and co-evolving ties. However, they pay relatively less direct attention to the role of recent historical opinions in bounded-confidence updating. In particular, their decaying effects remain less fully examined. This issue is especially relevant in contexts such as online consumption and information dissemination, where users’ current decisions are often jointly affected by current judgments, prior experiences, peer evaluations, and external guidance.

To address this issue, this paper proposes an extended HK-type opinion dynamics model that incorporates multiple information sources into the updating process. Specifically, each individual’s new opinion is jointly determined by current opinion, recent historical opinions, neighbors’ opinions, and a target opinion. Among these components, the historical-opinion term is constructed as a weighted average of recent historical opinions, and its influence is regulated by an attenuation rate, which reflects the gradual weakening of memory effects over time. The target opinion can be interpreted as an external directional signal, such as platform recommendation, advertising guidance, or authoritative information, while neighbors’ opinions represent the standard mechanism of social interaction. In this way, the proposed model is intended to better capture realistic opinion updating processes in which individuals balance self-persistence, recent memory, interpersonal influence, and external guidance, rather than relying only on contemporaneous neighbor interactions. This modeling design is consistent with the structure of the proposed update rule and with the practical scenarios discussed later in the paper, including e-commerce decision-making.

Compared with the above studies, the contribution of this paper is threefold. First, at the modeling level, it extends the HK framework by introducing a memory-based historical-opinion term with exponential attenuation, thereby providing a bounded-confidence model with explicit path dependence. Second, at the analytical level, it systematically investigates how the confidence threshold, attenuation rate, weighting coefficients, and network topology affect the emergence of consensus, polarization, and fragmentation. Third, at the application level, it discusses how the proposed model can be used to interpret consumer opinion evolution in e-commerce environments, where historical experience, peer reviews, and external guidance often act simultaneously.

The remainder of this paper is organized as follows. Section 2 introduces the model. Section 3 presents simulation experiments and parametric analyses. Section 4 discusses the application of the proposed model in e-commerce scenarios. Finally, Section 5 concludes the paper and outlines future research directions.

## 2. Model Description

### 2.1. Classical Bounded Confidence Models

The Deffuant–Weisbuch (DW) model [3] is proposed by Deffuant et al. At each step, a random pair of individuals interacts if their opinion difference falls within a confidence threshold. The opinion update rule is:

(1) $$\left\{\begin{array}{c} x_{i}\left(t+1\right)=x_{i}\left(t\right)+\mu_{ij}\left(t\right)\left[x_{j}\left(t\right)-x_{i}\left(t\right)\right]\\ x_{j}\left(t+1\right)=x_{j}\left(t\right)+\mu_{ji}\left(t\right)\left[x_{i}\left(t\right)-x_{j}\left(t\right)\right] \end{array}\right.$$

where $\mu_{ij}\left(t\right)$ denotes the influence weight of individual *j* on individual *i*.

The HK model [4] assumes each individual updates its opinion based on the opinions of neighbors within its confidence threshold. Let ε represent the bounded confidence threshold for individual *i*. The opinion update rule is:

(2) $$x_{i}\left(t+1\right)=\left\{\begin{array}{cc} \frac{1}{\left|\bar{N_{i}}\left(t\right)\right|}\sum_{j\in \bar{N_{i}}\left(t\right)}x_{j}\left(t\right), & \bar{N_{i}}\left(t\right)\neq ⌀\\ x_{i}\left(t\right), & \mathrm{otherwise} \end{array}\right.$$

where $\bar{N_{i}}\left(t\right)=\left\{j|\;|x_{i}\left(t\right)-x_{j}\left(t\right)|\leq \varepsilon ,\;1\leq j\leq N\right\}$ denotes the set of individuals within individual *i*’s confidence threshold.

Self-confidence parameters are introduced for (2) to reflect individual persistence. The MHK model [7] is proposed:

(3) $$x_{i}\left(t+1\right)=\left\{\begin{array}{cc} \lambda_{i}x_{i}\left(t\right)+\frac{1-\lambda_{i}}{\left|N_{i}\left(t\right)\right|}\sum_{j\in N_{i}\left(t\right)}x_{j}\left(t\right), & N_{i}\left(t\right)\neq ⌀\\ x_{i}\left(t\right), & \mathrm{otherwise} \end{array}\right.$$

where $N_{i}\left(t\right)=\{j|\;|x_{i}\left(t\right)-x_{j}\left(t\right)\left|\leq \varepsilon ,1\leq j\leq n,j\neq i\right\}$ and $\lambda_{i}\in \left[0,1\right]$ is the self-confidence parameter.

Recent extensions incorporate complex networks, emotional mobilization, and fuzzy logic [31,32,33]. Studies explore heterogeneous confidence thresholds (ε) and dynamic network topology, aligning with social psychology principles like selective exposure and cognitive dissonance.

### 2.2. Model Construction

Opinion evolution is not only affected by the opinions at present time, but also by the opinions at historical times. Let $x_{i}\left(t\right)$ denote the opinion of individual *i* at time *t*. The opinion update rule is:

(4) $$x_{i}\left(t+1\right)=\frac{\alpha x_{i}\left(t\right)+\beta H_{i}\left(t\right)+a_{i0}\left(t\right)\gamma T+\delta \cdot \frac{1}{|N_{i}\left(t\right)|}\sum_{j\in N_{i}\left(t\right)}x_{j}\left(t\right)}{\alpha +\beta +a_{i0}\left(t\right)\gamma +\delta \cdot 1_{|N_{i}\left(t\right)|>0}}$$

where ε∈[0,1] is the confidence threshold, and the coefficients α,β,γ,δ∈[0,1] represent the relative importance of the current opinion, historical opinions, target opinion, and neighbors’ opinions, respectively, and $1_{|N_{i}\left(t\right)|>0}$ is the indicator function, which equals 1 if the neighbor set is non-empty and 0 otherwise.

Regarding the denominator in the update rule, it is exactly the sum of the coefficients that actually contribute to the numerator. When a certain information source, for example the target opinion, is not activated because the confidence condition is not satisfied, the corresponding coefficient is removed from the denominator. Thus, the total weight always sums to one. Even when the target term is inactive, the update rule remains properly normalized and no systematic bias is introduced. Concerning the situation where an individual has no neighbor within its confidence threshold at a given time step, the neighbor term contributes nothing to the update, and the rule reduces to a form that depends only on the individual’s current opinion, historical opinions, and possibly the target opinion (if activated). This corresponds to an information-isolated state, where opinion change is driven only by internal memory and external guidance when accessible. Such a situation is meaningful in real social contexts.

T∈[0,1] is a predetermined target opinion, and

(5) $$a_{i0}\left(t\right)=\left\{\begin{array}{cc} 1, & |x_{i}\left(t\right)-T|\leq \varepsilon \\ 0, & \mathrm{otherwise} \end{array}\right.$$

$H_{i}\left(t\right)$ denotes the weighted historical average opinion for individual *i* at time *t*:

(6) $$H_{i}\left(t\right)=\sum_{k=t-3}^{t-1}{\bar{w}}_{k}x_{i}\left(k\right)$$

where

(7) $$w_{k}=e^{-\lambda \cdot (t-k)},\;S=\sum_{k=t-3}^{t-1}w_{k},\;{\bar{w}}_{k}=\frac{w_{k}}{S}$$

and λ∈[0,1] is the attenuation rate. Obviously, ${\bar{w}}_{t-3}\leq {\bar{w}}_{t-2}\leq {\bar{w}}_{t-1}$ and ${\bar{w}}_{t-3}+{\bar{w}}_{t-2}+{\bar{w}}_{t-1}=1$. As λ→0, $w_{k}=e^{-\lambda (t-k)}\to 1$ for k=t−3, t−2, t−1, so S→3 and ${\bar{w}}_{k}\to 1/3$; thus $H_{i}\left(t\right)$ reduces to the arithmetic mean of the three most recent opinions. As λ→∞, $w_{t-1}=1$, $w_{t-2},\;w_{t-3}\to 0$, hence S→1 and ${\bar{w}}_{t-1}\to 1$, ${\bar{w}}_{t-2},\;{\bar{w}}_{t-3}\to 0$; the model relies exclusively on the most recent historical opinion, $H_{i}\left(t\right)=x_{i}\left(t-1\right)$. In all cases S∈[1,3], ensuring numerical stability for any λ∈[0,∞).

**Figure 1:**
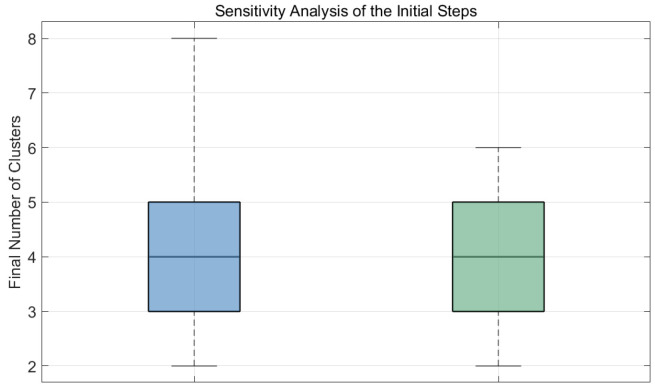
Sensitivity analysis of the initial steps.

As for the initialization of the historical-opinion term, the model uses a fixed memory window covering the three most recent time steps. During the first three steps, the historical window is not fully available. To ensure a stable start, we initialize the weighted historical average at t=0 as the individual’s initial opinion. From the next time step onward, the window is gradually filled with actual historical opinions, and the weighted average is computed as defined. This initialization scheme shows good numerical stability in all simulations and does not cause artificial jumps or distorted convergence. Similar treatments are commonly used in the existing literature on memory-driven opinion dynamics. To further examine whether the initialization of $H_{i}\left(t\right)$ during the first three steps affects the final clustering outcome, we additionally compare two different ways of handling the historical window. As shown in Figure 1, the blue boxplot represents the case where the historical window is completed using the initial opinion, whereas the green boxplot represents the case where only the currently available historical information is used and no additional filling is introduced when historical information is insufficient. The results show that the median, interquartile range, and overall variation of the final number of clusters are highly similar under the two treatments, indicating that the model is not particularly sensitive to the window-filling method in the initial steps. Therefore, the specific treatment adopted for t=0 to t=3 does not substantially alter the final clustering structure. With the above design, the proposed model remains mathematically self-consistent and behaviorally sound for all time steps and across all parameter configurations.

**Figure 2:**
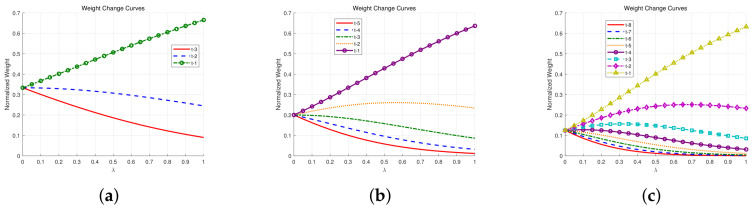
The variation of ${\bar{w}}_{k}$ with λ. (**a**) Three historical moments. (**b**) Five historical moments. (**c**) Eight historical moments.

Figure 2 shows that, the weight distributions under the three, five, and eight step memory windows exhibit similar overall trends. With an increasing attenuation rate λ, the weight of the most recent historical time t−1 rises significantly in all three cases, while the weights of earlier moments generally become smaller. Specifically, in Figure 2a, the weight of t−1 increases steadily, the weight of t−3 decreases substantially, and the weight of t−2 decreases only mildly. In Figure 2b, the weights of t−5 and t−4 decline rapidly and approach very small values, whereas the weight of t−2 first increases and then decreases slightly. In Figure 2c, a similar redistribution pattern can be observed: the earliest terms t−8 to t−5 quickly become negligible, while the intermediate terms t−2, t−3, and t−4 first increase and then gradually decline. These results suggest that, when the memory window is extended, the additional early historical terms contribute only limited information, especially for moderate and large λ.

The choice of a three-step historical window is grounded in both psychological principles and computational practicality. According to Ebbinghaus’s forgetting curve [34], human memory retention decays exponentially over time, following the form $R=R_{0}e^{-t/S}$. As shown in Figure 2b, when the attenuation rate λ exceeds 0.5, the normalized weights for time steps prior to t−3 approach zero, indicating that information beyond three historical steps exerts negligible influence on current opinion formation. Furthermore, cognitive psychology research on working memory capacity suggests that individuals can effectively retain approximately four discrete information units in working memory when rehearsal strategies are restricted [35]. For children, this capacity is even lower, approximately two to three items. Thus, the three-step window aligns with the inherent cognitive limitations of human information processing. This design choice avoids the unrealistic assumption that all historical opinions exert equal influence, while maintaining computational efficiency. It is also consistent with recent developments in memory-driven opinion dynamics models [36], which have independently recognized the importance of limited historical dependencies in modeling realistic opinion evolution.

### 2.3. Correspondence Between Model Parameters and Psychological Mechanisms

As shown in Table 1, each parameter in the proposed model is associated with a well-established psychological concept, clarifying the behavioral interpretation of the update rule.

## 3. Numerical Simulations

Simulation experiments with various parameters are conducted in order to explore the impacts of parameters on opinion evolution. Some representative experiments are presented in this section, which are helpful in revealing the underlying social–psychological mechanisms and providing supports for subsequent research and applications. To examine the role of network topology, we performed simulations on three distinct network structures, namely ER random networks, BA scale-free networks, and WS small-world networks. Due to space constraints, we present the results on the WS small-world network as a representative case. The WS network is selected because its combination of high clustering and short average path length facilitates both local opinion exchange and global consensus formation. At the same time, it avoids the local polarization often induced by hub nodes in BA networks and the structural irregularity of ER networks that tends to produce opinion fragmentation. Similar qualitative behaviors under parameter variations are observed across all three network types, and a detailed comparison is deferred to Section 3.4. Regarding population size, we tested populations of *N* = 100, 200, 500, and 1000. Results confirm that parameters drive identical convergence patterns, such as cluster count and convergence tendency. Therefore, we set *N* = 200 throughout to balance computational efficiency and visualization clarity. The target opinion is fixed at *T* = 0.8. Initial opinions are randomly and uniformly distributed within [0,1]. The confidence threshold ε and the attenuation rate λ are chosen randomly within [0,1].

### 3.1. Analysis of the Influence of Confidence Threshold on Opinion Evolution

**Figure 3:**
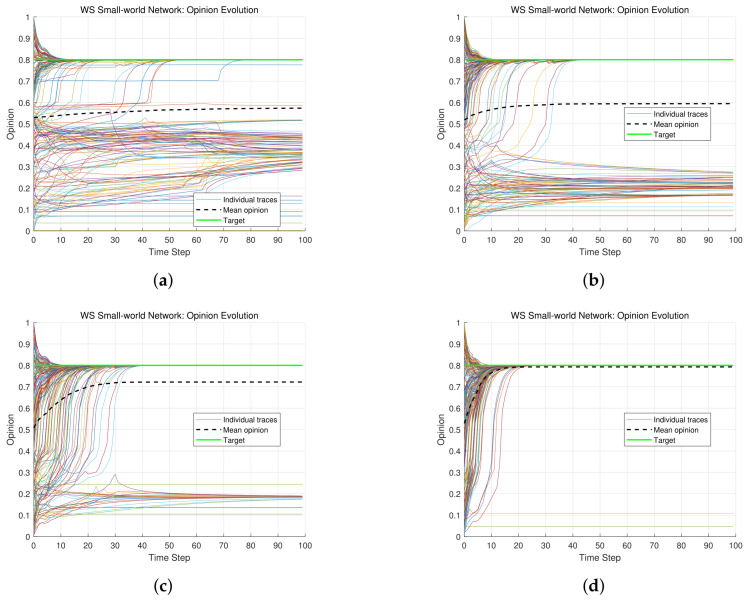
Opinion evolution under different confidence thresholds. (**a**) ε=0.2. (**b**) ε=0.3. (**c**) ε=0.4. (**d**) ε=0.5.

The confidence threshold is a core parameter that determines an individual’s psychological openness to new information. A low threshold indicates a conservative attitude, where an individual is only willing to accept information that aligns closely with their existing views. This psychological trait can easily lead to opinion fragmentation, as individuals tend to interact only with like-minded peers, effectively creating an “information cocoon.” Conversely, a high threshold signifies greater inclusiveness and adaptability, where the individual is willing to understand and accept a wider range of differing opinions. While this promotes faster information propagation and swift consensus, it also carries the risk of herd behavior, where individual judgment is suppressed by the majority opinion. Therefore, the confidence threshold directly influences how individuals establish connections within a network and fundamentally shapes the speed and scope of information diffusion. Some parameters are specified as λ=0.2, α=0.2, β=0.2, γ=0.4, δ=0.2. Let ε=0.2,0.3,0.4,0.5, respectively. The opinion trajectories of *N* individuals in these cases are shown in Figure 3.

In the case of ε=0.2, the opinion trajectories of 200 individuals are shown in Figure 3a The distribution of individual opinions exhibits obvious fragmentation, and multiple small-scale opinion clusters are formed among 200 individuals. In each cluster, individuals hold relatively consistent opinions and there is scarce interaction or communication between different clusters. A low confidence threshold implies that individual *i* (i=1,…,200) is only willing to communicate with the individuals whose opinions are very close to that of individual *i*. Hence, it is difficult for each individual to influence or be influenced by other individuals. Hence, the entire network splits into several isolated small groups (or clusters). Each group forms an opinion environment with a high degree of internal consistency, while there is a lack of effective communication channels between groups, which leads to a state of high-level polarization. This communication pattern reflects the selective exposure effect [37] in social psychology, namely, people tend to be exposed to the information that is consistent with their existing beliefs and avoid the information that may challenge their positions. In the case when the confidence threshold is too low, individuals are more likely to be trapped in an information cocoon [37] and only focus on specific types of information. Hence, a low confidence threshold tends to strengthen individuals’ existing opinions and reduces individuals’ openness to new information, which is not conducive to the exchange and development of multiculturalism.

In the cases of ε=0.3,0.4,0.5, although some opinion clusters still exist, the opinions of the 200 individuals gradually converge. In particular, as ε approaches 0.5, individuals begin to accept greater opinion differences, which facilitates extensive opinion communication and ultimately leads to a larger and more unified opinion distribution. A moderate confidence threshold allows individuals to flexibly assimilate new information, namely, individuals neither adhere to their original opinions rigidly nor abandon their positions hastily. This configuration encourages individuals to seek and consider diverse perspectives, enhance cross-cluster communication and promote opinion diffusion and integration. Furthermore, as more individuals engage in opinion communication, the former isolated subgroups begin to interact, which facilitates collective consensus-building. The cases of ε=0.3,0.4,0.5 are aligned with the cognitive dissonance theory in social psychology, which means that humans inherently seek internal harmony. Appropriately relaxing confidence thresholds can mitigate psychological discomfort from exposure to heterogeneous information, enable individuals to maintain self-identity and embrace new information. Such mechanisms are important for promoting social inclusivity, strengthening community cohesion, and nurturing tolerance for diversity and uncertainty.

When the confidence threshold is too high (e.g., ε=0.8), most individuals communicate and update their opinions more freely. The group rapidly achieves a highly homogeneous opinion distribution after a short period. However, extreme uniformity (termed the herd effect [37]) may emerge in certain scenarios. A high confidence threshold reflects individuals’ open acceptance of external information, accelerates information propagation and bridges even initially divergent opinions. Simultaneously, the absence of communication barriers creates an efficient information-sharing platform, which enables all individuals to access relevant information and evolve toward a shared direction collectively. In the long run, although high confidence thresholds promote efficient short-term collaboration, excessive uniformity may stifle individual creativity and uniqueness, lead to homogenization, and hinder long-term opinion evolution. Additionally, over-reliance on external validation may erode an individual’s own judgment, foster dependency on collective opinions and develop a behavioral pattern of blind conformity.

### 3.2. Analysis of the Influence of Attenuation Rate on Opinion Evolution

**Figure 4:**
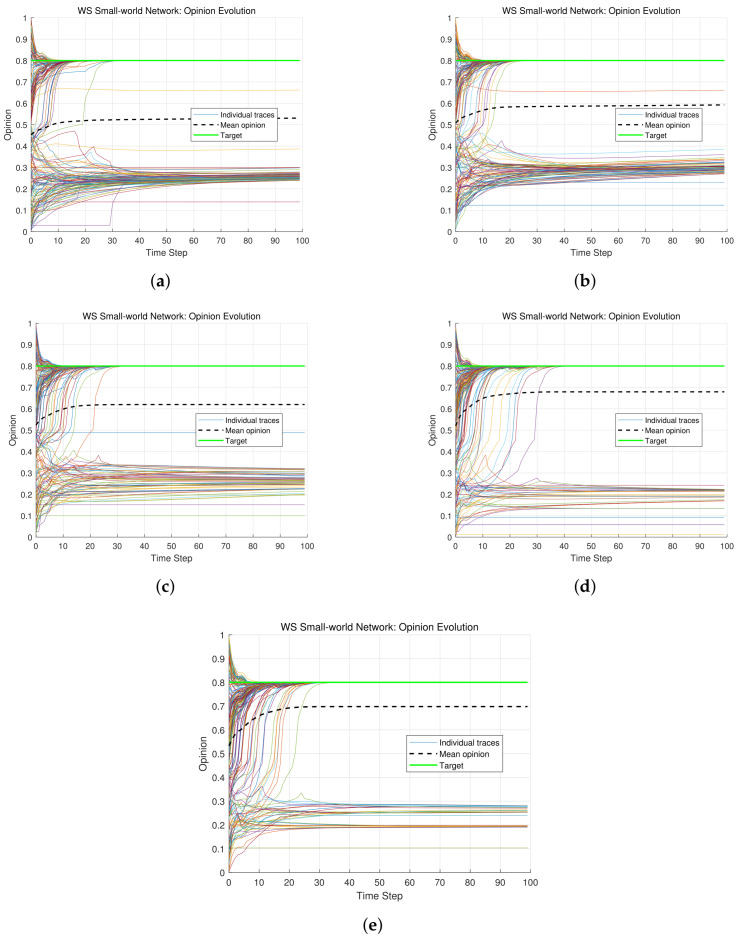
Opinion evolution under different attenuation rates. (**a**) λ=0.05. (**b**) λ=0.2. (**c**) λ=0.3. (**d**) λ=0.4. (**e**) λ=0.8.

The attenuation rate λ directly governs the weight distribution among an individual’s historical opinions. A larger λ causes the weight of the most recent historical opinion to dominate, while the weights of earlier opinions decay rapidly toward zero. Conversely, a smaller λ yields a more even weight distribution across the three historical steps. By tuning λ, the model can continuously vary the strength of historical path dependence: from strong reliance on a broad range of past opinions to nearly exclusive focus on the most recent experience. This flexibility allows the model to capture different memory patterns observed in real-world decision-making, ranging from long-term cognitive retention to short-term recency effects. The following simulations fix other parameters and systematically vary λ to isolate its impact on opinion evolution. Main parameters are specified as ε=0.35, α=0.2, β=0.2, γ=0.4 and δ=0.2. The opinion trajectories of *N* individuals for λ=0.05,0.2,0.3,0.4,0.8 are shown in Figure 4, respectively.

In the case of low λ (λ=0.05), the weights of historical opinions in $H_{i}\left(t\right)$ are nearly the same, namely, the influence degrees of past opinions on an individual are almost the same. From Figure 4a, it is found that opinion fusion is in a slow manner, and multiple opinion clusters are formed gradually. In the case of moderate λ (λ=0.2,0.3,0.4), there are some differences among historical opinion weights in $H_{i}\left(t\right)$. Hence, the differences among influence degrees of historical opinions on an individual are not very significant. There are still some opinion clusters in Figure 4b–d, while some opinion clusters begin to merge gradually. In the case of high λ (λ=0.8), there are quite obvious differences among historical opinion weights in $H_{i}\left(t\right)$. The weight at time t−1 is much greater than the weights at times t−2 and t−3, which means each individual is more concerned with the opinion at the recent historical time. From Figure 4e, individuals influenced by the target opinion converge towards the target quickly, while other individuals merge into a group. Obviously, the number of opinion clusters decreases as the attenuation rate increases.

Some sociopsychological mechanisms are revealed by the above simulation results. The case of low λ (less than 0.1) reflects cognitive rigidity [37]. That is, individuals adhere to historical experiences persistently, resist new information, and trap the group in an opinion deadlock. The case of moderate λ (0.2–0.4) embodies gradual adaptation [38]. In other words, individuals phase out outdated beliefs gradually, but still retain some historical dependence, which is consistent with real-world decision-making patterns characterized by stepwise preference adjustments. The case of high λ (more than 0.5) corresponds to short-term memory dependence [39]. That is, individuals focus on recent information solely, and shed historical constraints rapidly. This fosters impulsive decisions or herd effects [37], which are driven by transient influences rather than cumulative reasoning. These findings reflect the critical role of the attenuation rate in balancing historical inertia and adaptability.

### 3.3. Analysis of the Influence of Weighting Coefficient on Opinion Evolution

The weighting coefficients determine the proportional influence of four key factors on an individual’s final opinion: current opinion, weighted historical average opinion, external target opinion, and neighbors’ opinions. The assignment of these weights corresponds to an individual’s role in the social network. A high weight on one’s own opinion (current or historical) is characteristic of a leader or a conservative individual, emphasizing autonomy and resistance to external information. A high weight on neighbors’ opinions or the target opinion is characteristic of a follower, prioritizing collaboration, receptivity, and external guidance. By adjusting these coefficients, the model can simulate the behavioral patterns of different personality types, ranging from conservative and stubborn to easily guided and conformist. The specific combination of weights determines whether a group evolves slowly with persistent disagreements or converges rapidly towards a shared goal. After extensive simulations, the following representative weight combinations are selected:

(a) α=0.2, β=0.5, γ=0.1, δ=0.2
(b) α=0.2, β=0.2, γ=0.4, δ=0.2
(c) α=0.5, β=0.2, γ=0.1, δ=0.2

The other parameters are chosen as ε=0.35 and λ=0.4, randomly.

**Figure 5:**
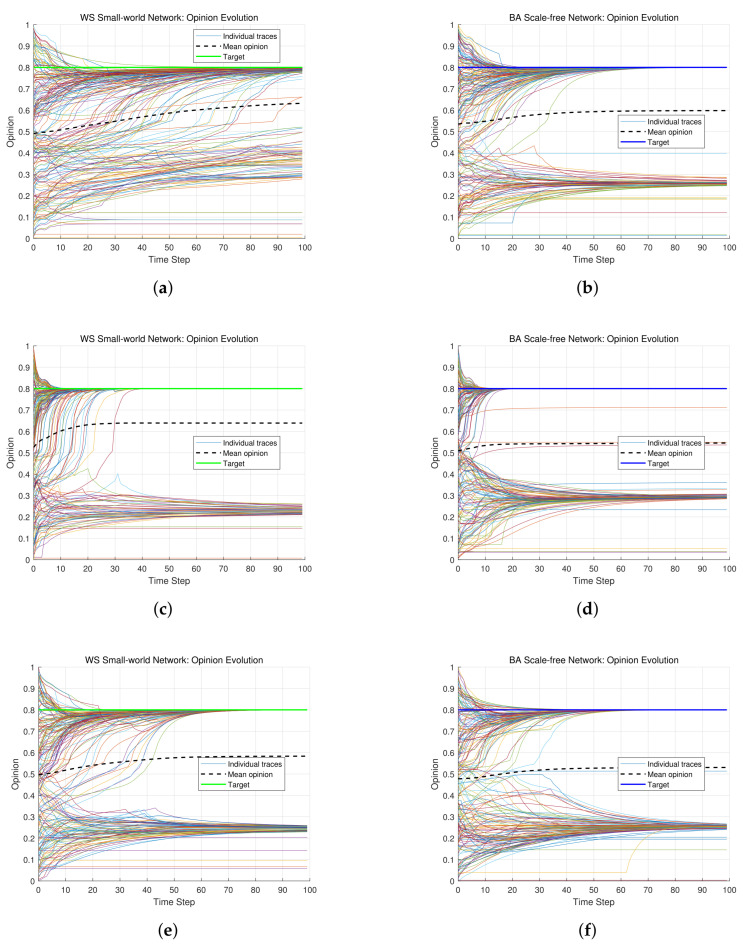
Opinion evolution under different weighting coefficients on WS small-world networks and BA scale-free networks. (**a**) High historical weight (WS network). (**b**) High historical weight (BA network). (**c**) Balanced weights (WS network). (**d**) Balanced weights (BA network). (**e**) High current weight (WS network). (**f**) High current weight (BA network).

In case (a), individuals assign higher weights to their historical opinions while paying less attention to the external target *T*. This indicates a preference for personal cognitive experiences and intuition over blind adherence to external suggestions. Consequently, the group exhibits a higher proportion of independent thinkers who are less susceptible to environmental changes. In Figure 5a,b, opinion distributions demonstrate strong conservatism, and multiple relatively isolated opinion clusters emerge. Moreover, the individuals in a cluster maintain internal consistency but diverge significantly from those in other clusters. Although some minor fusions occur, the overall dispersion remains high, which reflects individuals’ reliance on historical opinions. In this environment, although some new ideas may emerge, there is an overall tendency to stick to existing positions, which slows down the evolution of opinions and lead to persistent disagreements. The high weight on historical opinions reinforces cognitive stability and consistency, which results in slower individual responses to changes in other factors and makes it difficult for individuals to quickly accept and adapt to new environments.

By increasing the weight on the target opinion *T* and reducing dependence on historical opinions, opinion fusion is improved and accelerated, as shown in Figure 5c,d. Initially small and dispersed clusters gradually merge into larger and more cohesive clusters. Ultimately, a significant majority converges toward the target opinion *T* and demonstrate high consensus and coordination. Under this configuration, individuals prioritize collective goals and overarching trends, and have greater willingness to compromise or adapt for shared target. The increase of the weight on *T* improves openness to external information and facilitates cross-cluster interactions. Simultaneously, moderate historical opinion weights prevent excessive adherence to outdated beliefs, and enable individuals to strike a balance between retaining historical insights and assimilating new perspectives, which can avoid stubbornness and blind conformity.

In case (c), individuals assign a relatively higher weight to their current opinions (α=0.5). This indicates that individuals rely more heavily on immediate judgments in the process of forming new opinions. During initial iterations, the group exhibits moderate dispersion in opinions due to individuals’ adherence to current opinion, as shown in Figure 5e,f. Obviously, the dispersion in Figure 5e,f is decreased compared to that in Figure 5a,b. Both Figure 5a,b (β=0.5, high historical weight) and Figure 5e,f (α=0.5, high current weight) reflect strong individual reliance on personal opinions, which results in slow convergence and fragmented opinion distributions.

Some conclusions are obtained based on the simulation results for different weighting coefficients. In the case of high historical opinion weight (β=0.5), strong reliance on historical opinions makes individuals tend to maintain their existing positions when updating their opinions. This helps to enhance psychological stability in decision-making, but it also results in relatively delayed response to new information. In the case of high current opinion weight (α=0.5), individuals persistently adhere to immediate judgments but lack substantial guidance from the target opinion *T*. This tendency reinforces rigid adherence to individuals’ opinion, at the cost of innovation capabilities. For the balanced weight configuration (α=β=δ=0.2, γ=0.4), it provides an optimal equilibrium. Individuals not only retain partial dependence on historical opinions, but also evaluate new information rationally and adapt to emerging scenarios flexibly.

### 3.4. Analysis of the Influence of Network Topology on Opinion Evolution

In addition to individual parameters (e.g., confidence threshold, attenuation rate, weighting coefficients), the topology of social networks also profoundly shapes the final patterns of opinion evolution. To investigate the role of network structure, we compare three classic network topologies, ER random network (ER), WS small-world network (WS), and BA scale-free network (BA), under identical parameter settings (ε=0.35, λ=0.4, α=0.2, β=0.2, γ=0.4, δ=0.2). The initial opinions are uniformly distributed in [0,1], and the target opinion T=0.8. All simulations are conducted with N=200 individuals over 100 iterations.

**Figure 6:**
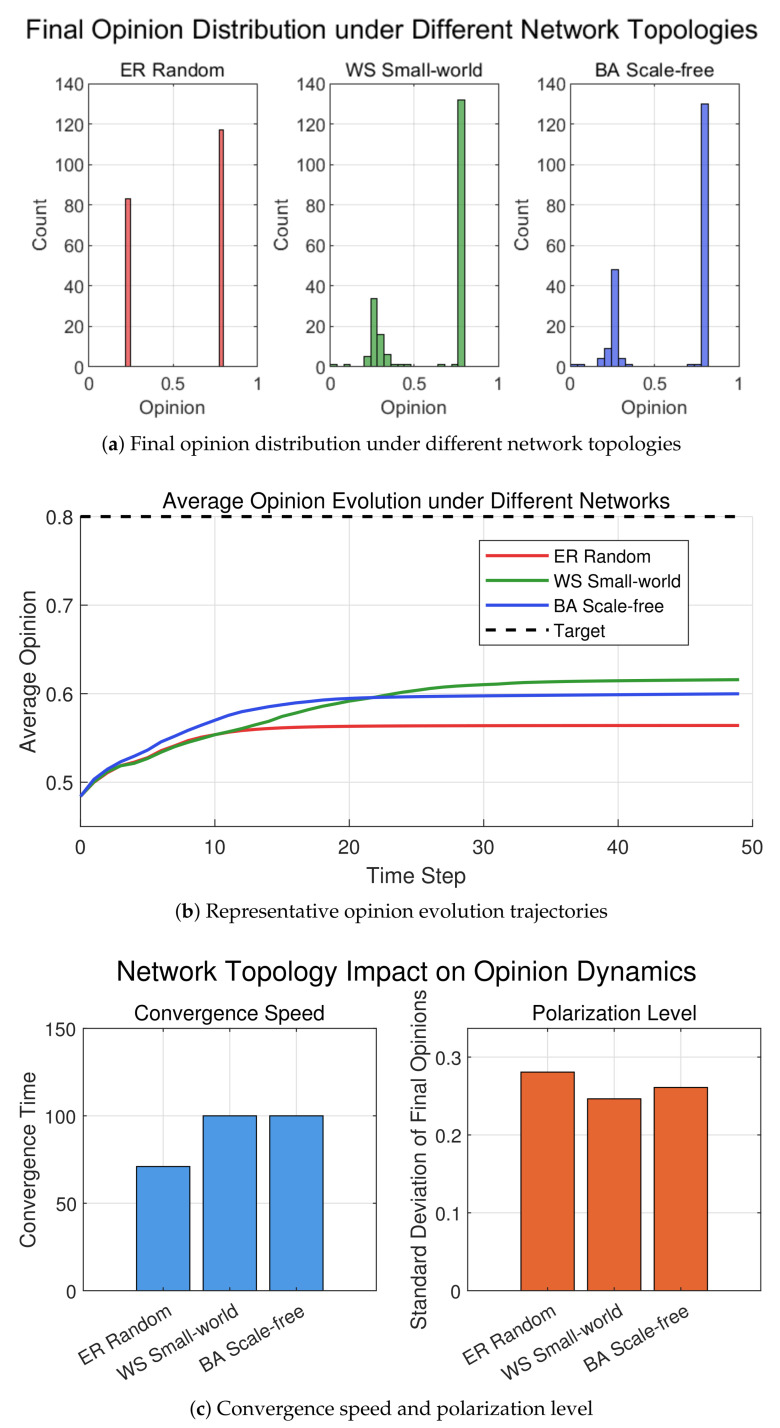
Comparison of opinion dynamics under ER random, WS small-world, and BA scale-free networks. (**a**) Histograms of final opinion distributions with standard deviations. (**b**) Typical opinion evolution processes (or distribution comparisons). (**c**) Convergence time and final standard deviation across networks.

Figure 6a presents the histograms of final opinion distributions under the three networks. The standard deviations are 0.2807 for ER, 0.2484 for WS, and 0.2610 for BA. The WS small-world network yields the most concentrated final opinion distribution (smallest standard deviation), indicating that its high clustering coefficient and short average path length facilitate local opinion exchange and global consensus formation. In the BA scale-free network, a few high-degree hub nodes accelerate opinion propagation but may also induce local polarization, resulting in a medium standard deviation. The ER random network, lacking structural regularities, leads to the most dispersed distribution and the highest polarization level.

Figure 6b shows representative opinion evolution trajectories (or distribution comparisons) under different networks. In the BA scale-free network, hub nodes (opinion leaders) quickly attract surrounding nodes, but differences among hubs may cause multiple clusters. In the WS small-world network, opinion evolution is smoother; clustering effects promote local consensus that gradually merges. In the ER random network, opinion changes are more disorderly and convergence is slow.

Figure 6c compares convergence speed and final polarization level (standard deviation) across the three networks. The BA scale-free network converges fastest due to hub-accelerated information diffusion, followed by the WS small-world network, while the ER random network is the slowest. Regarding polarization, the WS small-world network achieves the smallest final standard deviation, indicating its superiority in consensus building; the BA scale-free network is intermediate, and the ER random network exhibits the highest polarization.

In summary, network topology plays a critical role in opinion evolution. WS small-world networks excel at promoting consensus due to their high clustering and short path lengths. BA scale-free networks enable rapid information propagation via hub nodes but risk local polarization. ER random networks tend to produce fragmented opinions and slower convergence. These findings provide theoretical guidance for designing network interventions in real-world applications.

The simulations and analysis in Section 3.1, Section 3.2, Section 3.3 and Section 3.4 illustrate the roles of some key parameters on opinion evolution and reveal the hidden social and psychological laws, which can provide some guidance for designing appropriate parameters in different application scenarios.

## 4. Application Scenarios

In e-commerce environments, consumers’ purchase-related opinions are shaped by multiple sources of information, including product attributes, price comparisons, online reviews, brand reputation, previous shopping experience, and platform recommendations [40]. These factors make online consumption a typical scenario of multi-source opinion formation. Consumers do not make decisions only according to their current impressions of a product; rather, their judgments are also affected by past experiences, peer evaluations, and external promotional signals.

This setting is consistent with the structure of the proposed model. The current opinion represents consumers’ immediate product perception, the historical opinion term reflects the influence of previous experience, the neighbor-opinion term captures the effect of peer reviews and social interaction, and the target opinion can be interpreted as external guidance such as advertising, platform recommendation, or influencer promotion. Therefore, the e-commerce review scenario provides a suitable context for illustrating the practical applicability of the proposed model.

### 4.1. Simulation on a Real-World Social Network

To further examine the applicability of the proposed model in an e-commerce-related setting, we employ the real Epinions network as the interaction topology among consumers [41]. Epinions is a typical online consumer review platform, and its network provides an empirically grounded interaction topology for e-commerce environments. In the original dataset, the full network contains 75,888 individuals. Therefore, compared with artificially generated networks, this dataset can better reflect the structural characteristics of real online review communities.

**Figure 7:**
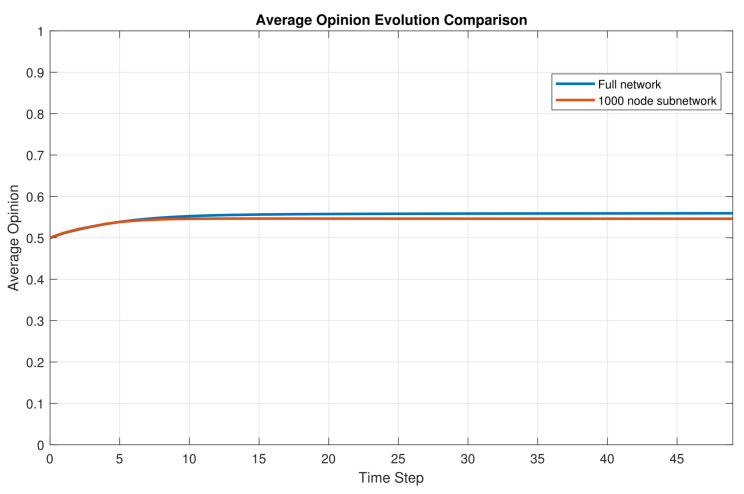
Comparison of average opinion evolution under different settings.

Before conducting the main simulations, we compare the average opinion evolution on the full Epinions network with that on a subnetwork consisting of the first 1000 individuals. As shown in Figure 7, the two curves are very close throughout the evolution process. This result suggests that the 1000-node subnetwork can preserve the main macroscopic evolutionary pattern of the full network. Considering both computational efficiency and visualization clarity, the following simulations are therefore conducted on the 1000-node Epinions subnetwork. Unless otherwise specified, the parameters are set as follows: N=1000, T=0.8, ϵ=0.35, λ=0.4, α=0.2, β=0.2, γ=0.4, δ=0.2.

In the baseline simulation of opinion dynamics, initial opinions are often assumed to follow a uniform distribution over [0,1]. This setting is useful as a neutral benchmark because it assumes that individuals’ initial opinions are evenly distributed across the whole opinion space. However, real online review data usually does not follow such an idealized distribution. In many e-commerce review systems, ratings tend to be concentrated around extremely high scores and, to a lesser extent, extremely low scores, forming a J-shaped distribution. Therefore, we compare two types of initial opinion distributions: a uniform distribution over [0,1] and a J-shaped distribution with stronger concentration at the two extremes.

**Figure 8:**
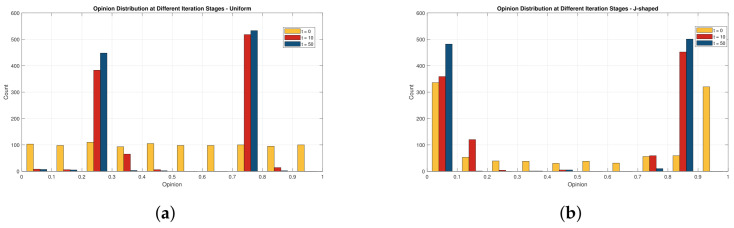
Distribution of individual (consumer) counts at different iteration stages. (**a**) Uniform initial distribution. (**b**) J-shaped initial distribution.

**Figure 9:**
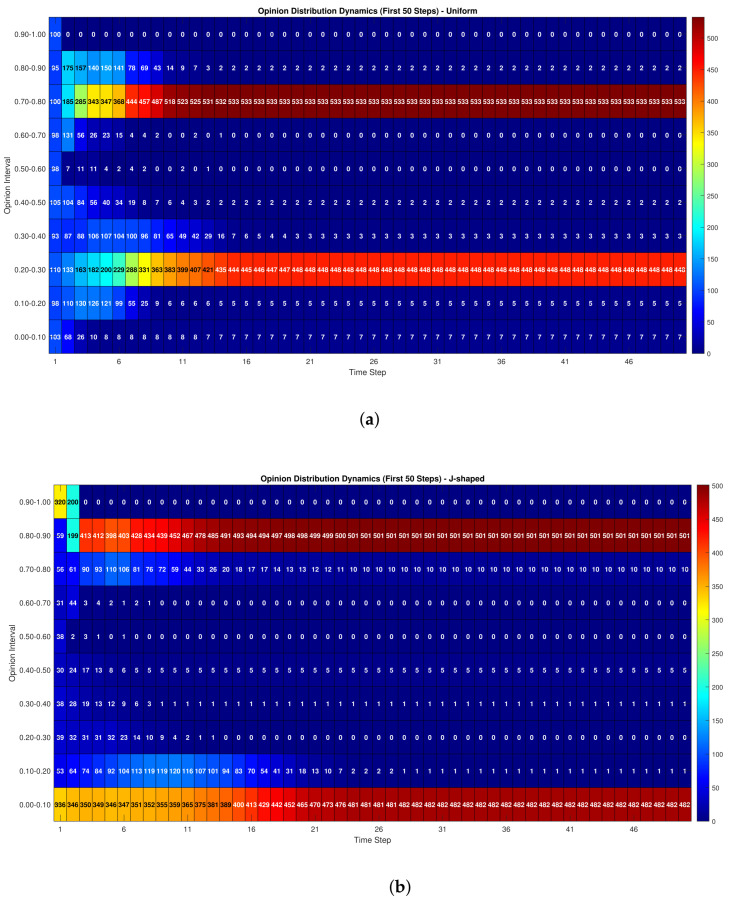
Temporal variations in individual (community) counts across iterations. (**a**) Uniform initial distribution. (**b**) J-shaped initial distribution.

Figure 8 presents the opinion distributions at t=0, t=10, and t=50 under two types of initial opinion distributions: uniform and J-shaped. In Figure 8a under the uniform initial distribution, opinions are initially scattered uniformly over [0,1]. As iterations proceed, the number of individuals close to the target opinion T=0.8 gradually increases, while the number far from the target decreases. This indicates that, under the combined influence of external guidance, peer interaction, and historical dependence, consumer opinions gradually move toward the promoted product perception. In Figure 8b under the J-shaped initial distribution, the initial opinions are more concentrated near the two ends of the interval, especially at the high-score end, which better reflects the rating bias commonly observed in online review platforms. Compared with the uniform case, the J-shaped distribution produces a stronger initial concentration of extreme opinions. Nevertheless, the subsequent evolution still shows a clear movement toward the target opinion. Figure 9a,b further show the temporal variations of opinion-interval counts during the first 50 iterations. Although the two initial distributions differ substantially at the beginning, their overall evolutionary tendencies remain broadly consistent after several iterations. This comparison indicates that the proposed model is not highly sensitive to the assumption of uniformly distributed initial opinions and remains stable when a more realistic J-shaped distribution is considered. This validates the effectiveness of the proposed model in simulating the dynamic changes of consumer opinions in e-commerce scenarios.

### 4.2. Influence of Weight Parameters on Consumer Behavior

If the weight of the current opinion (α) increases, individuals will rely more on their own opinions at the current moment when updating their opinions, and the relative dependence on historical opinions, neighbor opinions, and target opinions will decrease. This will result in relatively small changes in individual opinions and make them less susceptible to external factors. In the e-commerce scene, if consumers give too much weight to their current impression of product, then it is not easy to change their decisions (even if they receive positive reviews or significant discounts from other consumers). This may lead to an increase in the diversity of purchasing decisions within the group and make it difficult to form a unified purchasing trend.

If the weight of the weighted historical average opinions is high, the more individuals value past experiences and opinions, then the stronger the influence of historical opinions on current opinion updates. This may make individual opinion changes relatively conservative, and the impact of new information on them is relatively weak. In practical scenarios, if consumers have purchased similar products in the past and have had poor experiences, then consumers may find it difficult to change their views due to excessive reliance on historical experience, which affects the overall acceptance of the new product by the group and the formation of purchasing decisions.

If the weight of the target opinion is relatively high, then individuals are more likely to be attracted by the target opinion, adjust their opinions towards the direction of the target opinion, and are more likely to converge to the neighborhood of the target opinion. In e-commerce promotional activities, manufacturers can use marketing guidance, product advantage promotion, etc., to encourage consumers to change their views on products more quickly. That is, consumers shift from hesitant buying to inclined buying, which leads to an increase in group purchasing willingness, and group opinions evolve more quickly in a direction favorable to the purchase.

If the weight of neighbor opinions is high, then the opinions of an individual’s neighbors, such as evaluations and recommendations from other consumers, have a significant impact on the opinion update of the individual. Individuals are more susceptible to the influence of surrounding consumer opinions, and the role of information dissemination and mutual influence within a group is more obvious, which leads to more complex and diverse changes in group opinions. On e-commerce platforms, if consumers value the consumer experience of other consumers more, then they will be more inclined to purchase products when they receive many positive reviews. On the contrary, if a consumer receives a lot of negative reviews, even if the product itself has some attractiveness, then it is possible for the consumer to give up purchasing. Hence, group purchasing decisions undergo significant changes with the changes of neighbors’ opinions.

### 4.3. Attenuation Rate Regulation and Market Adaptability

The attenuation rate reflects the changing trend of the influence of historical opinions on current opinion. The larger the attenuation rate, the more consumers rely on information from the most recent historical moment; the smaller the attenuation rate, the stronger the consumer’s dependence on information from the past historical period. If the attenuation rate is large, then consumers may forget their initial impression of the product faster and are more easily influenced by new product promotion information or other recent consumer evaluations. Group opinions will be more sensitive to market changes. If the attenuation rate is small, then consumers may be influenced by their early views on the product for a long time, and their acceptance and response to new information may be relatively slow, which results in a lag in adjusting opinions.

### 4.4. Parameter Optimization Strategies and Risk Mitigation

In e-commerce scenarios, manufacturers can dynamically adjust the weight of different information based on marketing objectives. During the promotion period of new products, target weight can be increased to quickly establish market awareness through intensive advertising placement. During the user retention phase, historical weight can be moderately increased to maintain repeat purchase rates through brand loyalty. At the same time, it is necessary to enhance the weight of neighbors and amplify the word-of-mouth effect through user feedback and social communication.

However, we need to be vigilant about the potential risks of weight allocation. If consumers overly rely on historical opinions, it will be difficult for innovative products to overcome existing cognitive barriers. If the target weight monopolizes, it is possible to lead to information manipulation and damage to consumer rights. If neighbor interaction is ignored, the synergy of social networks will be weaken and the quality of group decision-making will be reduced.

The target opinion *T* embodies authoritative perspectives such as key opinion leaders in e-commerce environments, whose influence is dynamically activated through the conditional mechanism $a_{i0}\left(t\right)$. This selective engagement aligns with the bounded acceptance theory, where individuals assimilate external inputs proximate to their existing stance. To address such polarization, future work could explore two potential intervention strategies: enhancing relational ties by injecting social relationship weights to facilitate cross-cluster communication, and implementing dynamic parameter tuning to propel distant groups toward transitional opinion zones. These strategies, which remain underexplored in current research, offer promising avenues to mitigate polarization through targeted adjustments to social connections and dynamic cognitive processes.

## 5. Conclusions

Based on the HK model, this paper proposes an extended opinion dynamics model by considering the influence of historical opinions on opinion evolution. In the proposed framework, each individual’s opinion is determined not only by the opinions within its confidence threshold, but also by its recent historical opinions, whose influence decays over time. The effects of confidence thresholds, attenuation rates, weighting coefficients, and network topology on opinion evolution are explored through simulation experiments.

The simulation results show that low confidence thresholds tend to produce polarized or fragmented opinion patterns, whereas moderate thresholds facilitate opinion integration and consensus formation; excessively high thresholds may lead to rapid homogenization. The attenuation rate regulates the balance between historical dependence and responsiveness to new information. In terms of weighting coefficients, different configurations generate distinct convergence patterns. In addition, comparisons across ER random, WS small-world, and BA scale-free networks indicate that network topology has an important influence on convergence speed, final opinion distribution, and polarization level. To bridge the model parameters with psychological interpretations, we have provided a explicit mapping table linking confidence threshold, attenuation rate, and weighting coefficients to well-established social psychological mechanisms such as selective exposure, cognitive dissonance, recency effect, and herd behavior.

The proposed update rule can be written in matrix form as x(t+1)=W(t)x(t), where W(t) is a row-stochastic matrix. However, W(t) depends on the current opinion vector x(t) through the bounded-confidence condition $|x_{i}\left(t\right)-x_{j}\left(t)\right|\leq \epsilon$ and the target-activation condition $|x_{i}\left(t\right)-T|\leq \epsilon$. Hence W(t) is not a prescribed time-varying matrix but a state-dependent stochastic matrix. Analyzing the convergence of such state-dependent products is more challenging than for standard ergodic chains. A rigorous convergence analysis of such state-dependent dynamics remains an important direction for future theoretical work.

While a high attenuation rate λ accelerates consensus formation, this finding should not be interpreted as less memory is always beneficial for society. In real-world settings, historical memory serves as a critical filter against misinformation, fake news, and short-term manipulation. A population that rapidly forgets past opinions may become more vulnerable to external guidance (e.g., targeted advertising, political propaganda) and exhibit herd behavior. Therefore, the optimal λ should balance adaptability and stability, depending on the trustworthiness of the information environment.

Finally, by examining the proposed model on the real Epinions network in an e-commerce-related setting, we further illustrate the applicability of the framework on an empirically grounded interaction topology. However, this application should be regarded as an exploratory approximation rather than a complete representation of real consumer decision-making, since many practical factors are not explicitly included in the current model.

Overall, the main contribution of this study is to extend the HK framework by incorporating recent historical opinions and attenuation effects, and to show how memory, confidence, external guidance, and network topology jointly shape opinion evolution. Future work may further incorporate richer behavioral factors, more realistic empirical data, and additional network characteristics. In particular, the current model assumes a fixed target opinion *T* (set to 0.8) and a single external guidance source. Future extensions could consider dynamic targets that change over time or multiple competing targets (e.g., advertisements from different brands). To better handle such competitive scenarios, the opinion domain could be extended from [0,1] to [−1,1], allowing symmetric representation of opposing influences. These extensions would enable the study of market competition, opinion manipulation, and polarization dynamics in more realistic e-commerce environments.

## Figures and Tables

**Table 1:** Mapping between psychological mechanisms and model parameters.

Psychological Mechanism	Parameter	Role in the Model
Selective exposure	ε	Determines openness to dissimilar opinions
Cognitive dissonance	α, β	Resistance to change
Recency effect	λ	Balance between recent and remote memory
Herd effect	δ	Susceptibility to peer influence
Authority influence	γ	Responsiveness to external signals

## Data Availability

The simulation code supporting the findings of this study is available from the corresponding author upon reasonable request.

## References

[1] Rainer H., Krause U. (2002). Opinion dynamics and bounded confidence: Models, analysis and simulation. J. Artif. Soc. Soc. Simul..

[2] DeGroot M.H. (1974). Reaching a consensus. J. Am. Stat. Assoc..

[3] Deffuant G., Neau D., Amblard F., Weisbuch G. (2000). Mixing beliefs among interacting agents. Adv. Complex Syst..

[4] Lorenz J. (2007). Continuous opinion dynamics under bounded confidence: A survey. Int. J. Mod. Phys. C.

[5] Martins A.C. (2008). Continuous opinions and discrete actions in opinion dynamics problems. Int. J. Mod. Phys. C.

[6] Bernardo C., Altafini C., Proskurnikov A., Vasca F. (2024). Bounded confidence opinion dynamics: A survey. Automatica.

[7] Cheng C., Yu C. (2019). Opinion dynamics with bounded confidence and group pressure. Phys. A Stat. Mech. Appl..

[8] Cheng C., Yu C. (2022). Social conformity creates consensus and strong diversity of Hegselmann-Krause opinion dynamics. Sci. China Inf. Sci..

[9] Bernardo C., Vasca F., Iervolino R. (2021). Heterogeneous opinion dynamics with confidence thresholds adaptation. IEEE Trans. Control Netw. Syst..

[10] Fan K., Pedrycz W. (2015). Emergence and spread of extremist opinions. Phys. A Stat. Mech. Appl..

[11] Ioannidis E., Varsakelis N., Antoniou I. (2020). Promoters versus adversaries of change: Agent-based modeling of organizational conflict in co-evolving networks. Mathematics.

[12] Latané B. (1981). The psychology of social impact. Am. Psychol..

[13] Flache A., Mäs M., Feliciani T., Chattoe-Brown E., Deffuant G., Huet S., Lorenz J. (2017). Models of Social Influence: Towards the Next Frontiers. J. Artif. Soc. Soc. Simul..

[14] Chen G., Su W., Mei W., Bullo F. (2020). Convergence properties of the heterogeneous Deffuant–Weisbuch model. Automatica.

[15] Zhao Y., Kou G. (2016). Opinion evolution of a social group with extreme opinion leaders. Proceedings of the 2016 6th International Conference on Computers Communications and Control (ICCCC).

[16] Zhao Y., Kou G., Peng Y., Chen Y. (2018). Understanding influence power of opinion leaders in e-commerce networks: An opinion dynamics theory perspective. Inf. Sci..

[17] Chen J., Kou G., Wang H., Zhao Y. (2021). Influence identification of opinion leaders in social networks: An agent-based simulation on competing advertisements. Inf. Fusion.

[18] Ye M., Qin Y., Govaert A., Anderson B.D., Cao M. (2019). An influence network model to study discrepancies in expressed and private opinions. Automatica.

[19] Hou J., Li W., Jiang M. (2021). Opinion dynamics in modified expressed and private model with bounded confidence. Phys. A Stat. Mech. Appl..

[20] Dong J., Hu J., Zhao Y., Peng Y. (2024). Opinion formation analysis for Expressed and Private Opinions (EPOs) models: Reasoning private opinions from behaviors in group decision-making systems. Expert Syst. Appl..

[21] Li K., Liang H., Kou G., Dong Y. (2020). Opinion dynamics model based on the cognitive dissonance: An agent-based simulation. Inf. Fusion.

[22] Chen X., Tsaparas P., Lijffijt J., De Bie T. (2021). Opinion dynamics with backfire effect and biased assimilation. PLoS ONE.

[23] Cheng C., Luo Y., Yu C. (2020). Consensus for expressed and private opinions under self-persuasion. IFAC-PapersOnLine.

[24] Zhu J., Yao Y., Tang W., Zhang H. (2022). An agent-based model of opinion dynamics with attitude-hiding behaviors. Phys. A Stat. Mech. Appl..

[25] Tan E., Stemler T., Small M. (2024). Cognitive dissonance and introversion effects on opinion dynamics and echo chamber formation. Phys. A Stat. Mech. Appl..

[26] Dong Y., Zhan M., Kou G., Ding Z., Liang H. (2018). A Survey on the Fusion Process in Opinion Dynamics. Inf. Fusion.

[27] Wang C. (2022). Opinion Dynamics with Higher-Order Bounded Confidence. Entropy.

[28] Salahshour M. (2019). Phase Diagram and Optimal Information Use in a Collective Sensing System. Phys. Rev. Lett..

[29] Jędrzejewski A., Sznajd-Weron K. (2018). Impact of Memory on Opinion Dynamics. Phys. A Stat. Mech. Appl..

[30] Boschi G., Cammarota C., Kühn R. (2021). Opinion dynamics with emergent collective memory: The impact of a long and heterogeneous news history. Phys. A Stat. Mech. Appl..

[31] Xu H., Ai K., Cai H., Wu S., Xu M. (2020). AMHK: A novel opinion dynamics affection mobilization-based Hegselmann–Krause model. Proceedings of the 2020 IEEE International Conference on Systems, Man, and Cybernetics (SMC).

[32] Zhao Y., Xu M., Dong Y., Peng Y. (2021). Fuzzy inference based Hegselmann–Krause opinion dynamics for group decision-making under ambiguity. Inf. Process. Manag..

[33] Peng Y., Zhao Y., Hu J. (2023). On the role of community structure in evolution of opinion formation: A new bounded confidence opinion dynamics. Inf. Sci..

[34] Murre J.M., Dros J. (2015). Replication and analysis of Ebbinghaus’ forgetting curve. PLoS ONE.

[35] Cowan N. (2001). The magical number 4 in short-term memory: A reconsideration of mental storage capacity. Behav. Brain Sci..

[36] Liu Q., Chai L. (2023). The memory influence on opinion dynamics in coopetitive social networks: Analysis, application, and simulation. IEEE Trans. Control Netw. Syst..

[37] Abelson R.P. (1967). Mathematical models in social psychology. Advances in Experimental Social Psychology.

[38] Michael R.B., Garry M., Kirsch I. (2012). Suggestion, cognition, and behavior. Curr. Dir. Psychol. Sci..

[39] Friedkin N.E., Johnsen E.C. (1990). Social influence and opinions. J. Math. Sociol..

[40] Wan Y., Ma B., Pan Y. (2018). Opinion evolution of online consumer reviews in the e-commerce environment. Electron. Commer. Res..

[41] Richardson M., Agrawal R., Domingos P. (2003). Trust Management for the Semantic Web. Proceedings of the The Semantic Web–ISWC 2003.

